# Supplementary material for: A locus conferring tolerance to Theileria infection in African cattle
Source: PLoS Genet. 2022 Apr 21;18(4):e1010099. doi: 10.1371/journal.pgen.1010099 (PMC9022807; doi:10.1371/journal.pgen.1010099)
Supplement: Attachment — Submitted filename: 2021_Plos Genetics Review.docx [file pgen.1010099.s008.docx]

In this interesting and important study, a genetic analysis of cattle which demonstrate heritable tolerance to infection by T. parva (the cause of East Coast Fever, ECF) was performed. Evidence has accumulated over many years to suggest that the frequency of natural tolerance alleles differs between cattle from endemic and non-endemic areas. The current study is the first to map genetic loci driving tolerance to ECF. Current treatment and prevention measures to control ECF are imperfect and expensive. The discovery of a genetic marker to aid selectively breeding tolerant cattle has huge economic significance. 4 genes associated with the variants conferring tolerance; two olfactory receptors (OR51H1 and OR51G1), one paralog of haemaglobin subunit beta (HBB) and one paralog of FAS-associated factor 1 (FAF1). Because T. parva infected cells are resistant to FAS-induced cell death, FAF1 was considered for further analysis. To test the effect of tolerant (T/T) vs reference (C/C) FAF1B alleles on in vitro expansion of *T. parva* infected cells, PBMCs were isolated from 12 cattle with the FAF1B T/T (tolerant) and 12 with the C/C (susceptible) genotype. For the 12 T/T cattle, 6 were from tolerant pedigree, and 6 were from unrelated cattle. Cells from the C/C genotype exhibited significantly increased growth rate (as counted by manual counting of cells stained with trypan blue) and parasite infection (as measured by staining the parasite with fluorescently labelled antibodies against the parasite surface, PIM).

As the editor knows, I am not an expert in genetics, and cannot critically review the genetic analysis tools applied. I therefore focus my review on the figure 5 and the overall impact. I do however also have a few small comments and questions about the genetic linkage part of the study.

Table 1: what is the explanation for the rather high proportion of cattle deaths in sire 3167 offspring 2018 study (both 2017 and 2018 studies involved 2^nd^ generation offspring). Why are the 2017 and 2018 studies so different?

Line 139: could you explain a bit more clearly why the 29 animals from the 2018 study were selected (where only 30% of sire 3167 survived).

A genomic region significantly associated with survival outcome was identified. 58066 variants, and 235 genes in the region were identified. 55 variants were selected for study. Can you explain what criteria were used to select the 55 variants predicted to have a functional consequence? (line 153). The authors state in the method that Ensembl’s variant effect predictor was used. As a non-geneticist I wondered what this means.

Figure 5: This experiment nicely supported the observation made that PBMCs isolated from cattle that were homozygous for the “reference” FAF1B SNP (C/C) were more highly infected with T. parva (as shown by staining with the schizont surface marker PIM), and correspondingly exhibited increased cell proliferation. Conversely cells isolated from animals that were homozygous for the “tolerant” SNP (T/T) were less susceptible to infection and consequently underwent less proliferation. These experiments were well controlled by using same number of PBMCs and normalizing the number of sporozoites obtained from the ticks by counting infected acini. Importantly cells from C/C and T/T cows were infected with the same batch of sporozoites, meaning that variations in sporozoite infectivity is less likely to impact the data.

It would be great to see some further functional validation of the potential role of FAF1B mutations in driving the tolerance phenotype. The authors already point out that FAF1B expression could not be detected in blood either before or after infection (pre-empting an obvious question and making it more difficult to imagine that FAF1B in fact does play an important role). It would indeed (as the authors already state) be important to check for FAF1B expression in other tissues.

I can think of a number of experiments that would be interesting to perform. One suggestion is to test the susceptibility of FAF1B C/C vs T/T cells before and after infection to Fas mediated apoptosis. A second experiment worth considering is to use CRISPR/Cas9 to introduce the FAF1B “tolerant” SNP (or otherwise knock out or knock down FAF1B) in cells isolated from susceptible cattle and then test their susceptibility to infection. This would be a great way to test whether it really is the SNP in FAF1B that drives tolerance.

However, I do very much appreciate the precise way the authors present their work, they do not attempt to over-sell or over-state their findings (e.g. line 312: “….. further worked required to elucidate any causal relationship between the FAF1 paralogue and enhanced tolerance…….”, and thus in my opinions additional experiments are not a requirement for this publication.
