# Supplementary material for: A locus conferring tolerance to Theileria infection in African cattle
Source: PLoS Genet. 2022 Apr 21;18(4):e1010099. doi: 10.1371/journal.pgen.1010099 (PMC9022807; doi:10.1371/journal.pgen.1010099)
Supplement: Attachment — Submitted filename: ECF-response.docx [file pgen.1010099.s009.docx]

Editor:

1) Provide a detailed list of your responses to the review comments and a description of the changes you have made in the manuscript.

*This document.*

2) Upload a Striking Image with a corresponding caption to accompany your manuscript if one is available (either a new image or an existing one from within your manuscript). If this image is judged to be suitable, it may be featured on our website. Images should ideally be high resolution, eye-catching, single panel square images. For examples, please browse our [archive](https://journals.plos.org/plosgenetics/volume). If your image is from someone other than yourself, please ensure that the artist has read and agreed to the terms and conditions of the Creative Commons Attribution License. Note: we cannot publish copyrighted images.

*We have provided an image of the founding sire (3167) of the study.*

3) In your filtering pipeline, you only considered variants with "high impact" according to VEP prediction. This seems overly stringent to me. I understand the rationale of prioritizing protein coding variants. However, there are arguably much more missense variants with detrimental effects on protein function than "high impact" variants in the genome. Please expand your analysis to include "moderate impact" variants. If you include the "moderate impact" variants, will this lead to more than 4 candidate causal variants?

*Applying the less stringent impact filter to retain both moderate and high impact variants, followed by the allele frequency filtering, returns a further 48 (missense) variants. As well as now reporting these in the main text (lines 156-166) they can now also be found in revised supplementary table 6 that enables the reader to filter on impact (i.e. ‘moderate’ and ‘high’), in addition to population-level allele frequencies (i.e. ‘Boran-(Pedigree)’ > 0.5 and ‘European-taurine’ < 0.1). We believe due to its high impact and the known role of FAS in T. parva infection the studied variant remains the strongest candidate in this region.*

4) Please provide Table S6 in a format that allows easy filtering and sorting of the variants (e.g. as an Excel file).

*Supplementary Table S6 has now been reformatted for ease of filtering as detailed above.*

Reviewer 1:

The functional effect of the loss-of-function variant remains unclear. Possible Faf1 knockout mouse model are not mentioned. Obviously Faf1 plays a key role in the dopaminergic neurodegeneration according to DOI: 10.1093/hmg/ddt006.

*Thanks. A complication of the use of this mouse model with respect to the present study is that the mouse genome doesn't actually carry the FAF1B gene (a BLAT search of the FAF1 paralog sequence against the mouse genome returns only hits to the FAF1 gene). So as T.parva isn’t able to infect mice, and as mice do not carry FAF1B, it may be difficult to draw direct comparisons to the cow results using this model.*

It appears to be a Mendelian trait as TT homozygotes are at an advantage but there is also an effect in heterozygous carriers: which type of inheritance best explains this?

*Yes, our data suggests an additive mode of inheritance, as indicated by the significant additive effect of 0.43 (t-statistic = 5.2; p = 2.51 x 10^-06^) for each copy of the T allele at the FAF1B genotype. This is further exemplified in results such as Figure 4 that show heterozygotes sit between the homozygotes on various metrics.*

In general, susceptibility/resistance against infectious diseases represents a complex trait of polygenic nature. Therefore, the question arises as to what proportion of the total variance this specific variant explains. Whether this finding provides full protection against natural infection is not completely clear.

*The SNV is estimated to explain 31.9% of the phenotypic variance in these cohorts, and this has now been reported in the manuscript at line 245.*

The option mentioned in the conclusion to spread this beneficial allele by selection or even targeted genome editing seems questionable to me, as this requires a targeted breeding program and the use of artificial insemination of appropriate bulls with the desired FAF1 genotype.

*We have developed a simple assay to genotype the FAF1B locus and are already working with our partners at ILRI and the African Dairy Genetics Gains project to explore how best to integrate this finding into their ongoing East African breeding programs. We agree there will be challenges, and are for example validating that selection on the locus will not have adverse impacts on production traits, but we do not envisage any insurmountable hurdles regarding integrating this locus into their current breeding strategies. Targeted breeding programs are possible in the T. parva-endemic regions, especially within the dairy population. Artificial insemination is also not uncommon in these areas. One of the major activities of the Centre for Tropical Livestock Genetics and Health, where much of this work was undertaken, is the establishment of genome editing technologies in both cattle and poultry. As such there is a natural flow to these technologies within the Centre.*

The indicine nature of the Boran breed could be mentioned once at the beginning.

*The indicine nature of Boran cattle is now mentioned in the abstract (Line 21), author summary (line 35), introduction (line 72) and results (lines 77-78), .*

The nonsense variant in FAF1 should be mentioned more clearly, at least in the summary and maybe even in the title.

*Thanks. That a nonsense variant in a paralogue of FAF1 is associated with survival is now highlighted in the abstract (line 25) and author summary (line 38).*

The International Society for Animal Genetics (ISAG) desires the use of human gene and variant nomenclature rules for domestic animals. The gene names should adhere to HGNC (www.genenames.org/).

*All genes listed are using the approved HGNC nomenclature.*

According to HGVS recommendations, the term “polymorphism” should be exchanged with “variant” (see <https://varnomen.hgvs.org/>).

*Instances of ‘polymorphism’ have been changed to ‘variant’, and ‘SNP’ has been changed to ‘SNV’ throughout.*

Please adhere to the published guidelines of sequence variant nomenclature (http://varnomen.hgvs.org/) e.g. the GenBank accessions of the used mRNA and protein entries should be mentioned to understand the given positions.

*All variants are described in detail in Supplementary Table 6, including the precise genomic location, gene and transcript IDs, and where relevant the cDNA, CDS and protein positions along with amino acids and codons. We have added to the main text at line 198 the appropriate sequence variant nomenclature for the FAF1B SNV (NC_037342.1:g.50823230C>T).*

The discussion turns out to be relatively short, conspicuously little literature is cited. For example a cross comparison to the very few known similar major gene effects against infectious diseases in domestic animals, e.g. TMEM154-related SRLV-susceptibility in sheep could enrich the discussion.

*Thanks. The opening paragraph to the discussion now highlights some of the literature reporting naturally occurring variants that confer resistance to disease in livestock (lines 302 to 310).*

Figure 1 might be moved into the supplement as it shows very detailed phenotypic records but lacking the individual FAF1 genotypes.

*A key motivation for Figure 1 was to highlight how all animals were showing signs of infection. Across all field trials, and both the controls and offspring of 3167, the same elevated temperature at around day 15 is consistently observed, indicative of infection. We believe this is an important point with regards to the downstream analyses as it illustrates that those animals that did not succumb to infection were not likely just uninfected. As it also shows the breakdown of outcomes by trial, sire etc., our preference would be to maintain Figure 1 in the main manuscript if possible.*

The very recent run9 variant catalogue of the 1000 Bulls genome Project includes also many indicine cattle genomes, maybe this resource could be consulted to improve the presentation of the allele distribution within taurine and indicine cattle.

*Thanks for the suggestion. We attempted to extract these data from the 1000 bulls files on SRA, however, it turns out to be difficult to identify and analyse specific breeds/sub-species in the data as this information is not included in the VCF files. We did though only use European taurine samples in the filtering of the variants for which we had 145 animals spanning 14 breeds, so we believe enough to be confident whether a variant is largely absent from European taurine animals. We now have specified the number of samples per sub-population at lines 192-193.*

I wonder if there were attempts to look for structural variants in the sequenced genomes.

*An initial exploration for structural variants (SVs) was performed on animals for which short-read sequence data was generated using Delly (*[*https://github.com/dellytools/delly*](https://github.com/dellytools/delly)*). Deletions (DEL), duplications (DUP), inversions (INV) and translocations (TRA) were called, and subsequently genotyped across all animals. A Fisher’s exact test on SV genotypes per survival outcome in the pedigree animals was subsequently performed, and no SVs on chromosome 15 were found to be significantly associated with survival. A sentence to this effect has been added at line 133.*

*Below are plots of the -log10(P) values from the Fisher tests for each SV type. Short-read sequence data is though potentially unreliable for studying structural variants - which is further complicated by the divergence between the European taurine reference and these African indicine samples. Given that no significant SV was detected on the chromosome on which we mapped an association with ECF tolerance, further exploration of SVs was discontinued. We hope in the future to apply long-read sequencing to assemble individuals from the pedigree in order to investigate structural variation more accurately.*

Reviewer 2:

Table 1: what is the explanation for the rather high proportion of cattle deaths in sire 3167 offspring 2018 study (both 2017 and 2018 studies involved 2nd generation offspring). Why are the 2017 and 2018 studies so different?

*As the field trials are natural challenge experiments, they are subject to inevitable temporal variation in T. parva strain, prevalence and infectivity. We believe that the variability in mortality observed between experiments is likely partly a result of these natural variations, and is why we fitted trial as a covariate, i.e. to try and account for these natural environmental fluctuations.*

Line 139: could you explain a bit more clearly why the 29 animals from the 2018 study were selected (where only 30% of sire 3167 survived).

*The decision to collect samples for the RNA study was made after the 2017 experiment. Thus, the 29 animals selected were the full complement of animals challenged in the final 2018 study (see Table 1). The animals for both the 2017 and 2018 studies were selected in a similar fashion. We identified progeny of male offspring of 3167 through breeding records and subsequent confirmation by genotype analysis. Unrelated animals were also selected from breeding records and by genotype analysis. As far as possible the animals were age-matched and were used shortly after weaning to be of a size suitable for handling in Ol Pejeta. All animals were screened by serology and PCR for tick-borne disease as indicated in the paper. It is important to note that the animals were not selected according to the FAF1B genotype, as this had not been identified at the time the studies were undertaken.*

A genomic region significantly associated with survival outcome was identified. 58066 variants, and 235 genes in the region were identified. 55 variants were selected for study. Can you explain what criteria were used to select the 55 variants predicted to have a functional consequence? (line 153). The authors state in the method that Ensembl’s variant effect predictor was used. As a non-geneticist I wondered what this means.

*Ensembl’s Variant Effect Predictor (VEP) indicates the impact of each variant according to its predicted functional consequence. A table summarising variant sequence ontologies and impacts is available at Ensembl (*[*https://m.ensembl.org/info/genome/variation/prediction/predicted_data.html*](https://m.ensembl.org/info/genome/variation/prediction/predicted_data.html)*). In addition to its mention in the methods, the main text and caption for figure 3 have been updated to explicitly highlight that VEP was used (lines 155 and 189).*

Figure 5: This experiment nicely supported the observation made that PBMCs isolated from cattle that were homozygous for the “reference” FAF1B SNP (C/C) were more highly infected with T. parva (as shown by staining with the schizont surface marker PIM), and correspondingly exhibited increased cell proliferation. Conversely cells isolated from animals that were homozygous for the “tolerant” SNP (T/T) were less susceptible to infection and consequently underwent less proliferation. These experiments were well controlled by using same number of PBMCs and normalizing the number of sporozoites obtained from the ticks by counting infected acini. Importantly cells from C/C and T/T cows were infected with the same batch of sporozoites, meaning that variations in sporozoite infectivity is less likely to impact the data.

It would be great to see some further functional validation of the potential role of FAF1B mutations in driving the tolerance phenotype. The authors already point out that FAF1B expression could not be detected in blood either before or after infection (pre-empting an obvious question and making it more difficult to imagine that FAF1B in fact does play an important role). It would indeed (as the authors already state) be important to check for FAF1B expression in other tissues.

I can think of a number of experiments that would be interesting to perform. One suggestion is to test the susceptibility of FAF1B C/C vs T/T cells before and after infection to Fas mediated apoptosis. A second experiment worth considering is to use CRISPR/Cas9 to introduce the FAF1B “tolerant” SNP (or otherwise knock out or knock down FAF1B) in cells isolated from susceptible cattle and then test their susceptibility to infection. This would be a great way to test whether it really is the SNP in FAF1B that drives tolerance.

However, I do very much appreciate the precise way the authors present their work, they do not attempt to over-sell or over-state their findings (e.g. line 312: “….. further worked required to elucidate any causal relationship between the FAF1 paralogue and enhanced tolerance…….”, and as such I would not insist on any additional experiments.

*Many thanks for These excellent suggestions. We have now expanded the discussion to cover these points.*
